# Next Generation Infectious Diseases Monitoring Gages via Incremental Federated Learning: Current Trends and Future Possibilities

**DOI:** 10.1155/2023/1102715

**Published:** 2023-03-01

**Authors:** Iqra Javed, Uzair Iqbal, Muhammad Bilal, Basit Shahzad, Tae-Sun Chung, Muhammad Attique

**Affiliations:** ^1^Department of Software Engineering, National University of Modern Languages, Islamabad 44000, Pakistan; ^2^Department of Artifical Intelligence and Data Science, National University of Computer and Emerging Sciences, Islamabad 44000, Pakistan; ^3^Department of Computer Science, National University of Computer and Emerging Sciences, Islamabad Chiniot-Faisalabad Campus, Chiniot 35400, Pakistan; ^4^Department of Artificial Intelligence, Ajou University, Suwon-Si 16499, Republic of Korea; ^5^Department of Software, Sejong University, Seoul 05006, Republic of Korea

## Abstract

Infectious diseases are always alarming for the survival of human life and are a key concern in the public health domain. Therefore, early diagnosis of these infectious diseases is a high demand for modern-era healthcare systems. Novel general infectious diseases such as coronavirus are infectious diseases that cause millions of human deaths across the globe in 2020. Therefore, early, robust recognition of general infectious diseases is the desirable requirement of modern intelligent healthcare systems. This systematic study is designed under Kitchenham guidelines and sets different RQs (research questions) for robust recognition of general infectious diseases. From 2018 to 2021, four electronic databases, IEEE, ACM, Springer, and ScienceDirect, are used for the extraction of research work. These extracted studies delivered different schemes for the accurate recognition of general infectious diseases through different machine learning techniques with the inclusion of deep learning and federated learning models. A framework is also introduced to share the process of detection of infectious diseases by using machine learning models. After the filtration process, 21 studies are extracted and mapped to defined RQs. In the future, early diagnosis of infectious diseases will be possible through wearable health monitoring cages. Moreover, these gages will help to reduce the time and death rate by detection of severe diseases at starting stage.

## 1. Introduction

At the end of 2019, the infectious disease, coronavirus, broke out in China and spread across the globe in a few months. The World Health Organization (WHO) declared that COVID-19 (Coronavirus Disease-19) is a deathly pandemic and resulted in different sorts of challenges around the world [1]. Although the patterns are still clear, studies indicate that this major issue will continue to exist over the next few years. COVID-19 is a general infectious disease that affects the human respiratory system. One of the general infectious diseases is SARS (severe acute respiratory syndrome), influenza, and cold viruses, which are well-known. Furthermore, despite being exposed to these diseases, only a small percentage of the population produces antibodies, according to surveys conducted in various nations. This proves that most patients will regularly require examinations by a limited number of doctors in short intervals due to resource constraints. Infectious diseases are usually diagnosed by using at least one of these three tests: chest X-ray, RT-PCR (reverse-transcriptase polymerase chain reaction), and computed tomography.

In sputum or a nasopharyngeal sample, the RT-PCR assay detects viral RNA (ribonucleic acid). It requires the use of specialist materials and equipment that are not widely available, and it typically inconveniently takes 12 hours because patients with an infectious disease must be identified and monitored as quickly as possible. Tests that use RT-PCR to determine results performed on the same patients at different times throughout the illness were found to be inconsistent, resulting in a high false-negative rate [2]. CT scan and 3D radiography images from intelligent diagnostic devices are used in a variety of clinical perspectives. Most hospitals lack the necessary equipment for this process. Patients are observed and treated on the base of clinical history. The equipment required for this examination in CXR (chest X-ray) is less cumbersome and easily adjusted. These resources are, for the most part, effortlessly accessible [3].

With the rapid evolution of electronic health records, it is now easier to use data for predictive modelling and subsequent advancements. Different applications and approaches in healthcare involve distributed machine learning, including electronic health records and chatbots, to detect a pattern in clinical status, detect the type of cancer treatment, and identify unusual diseases or infections and pathology. Contactless COVID-19 patient identification is carried out through the classification of COVID-19 cough samples, and the detection of these symptoms is accomplished by using advanced algorithms and procedures, resulting in more relevant, tailored, and accurate patient care. In addition, sensors are introduced that both monitor the temperature with facial recognition and upload each person's record to a directory [4]. Organizations are increasingly focusing on developing more efficient algorithms and using the potential of deep learning to build acceptable solutions in tackling exact, real-world challenges in the health sector.

To overcome the challenges of patients who are unaware of their symptoms at the first stage of the disease or who cannot go for a regular check-up for many reasons, DL can be used to analyse electronic health records. Due to its transformative potential, DL is a subset of ML (machine learning) and AI (artificial intelligence) that adds a new layer of complexity to medical technology solutions. The healthcare industry is using DL efficient records with efficiency and exceptional speed [5]. The modern healthcare system is extremely helpful, which makes prediction processes fast, efficient, and accurate with good learning ability, and more benefits lie within the neural networks formed by using AI and ML. The design and working of DL neural networks are like the system of the human brain. Because of multilayer networks and technology, it can be easily managed and sifted through vast quantities of data that would be lost or missed. Networks in deep learning can solve complex problems and can handle reams of data, which is very helpful in the profession of healthcare and federated learning [6].

**Figure 1:**
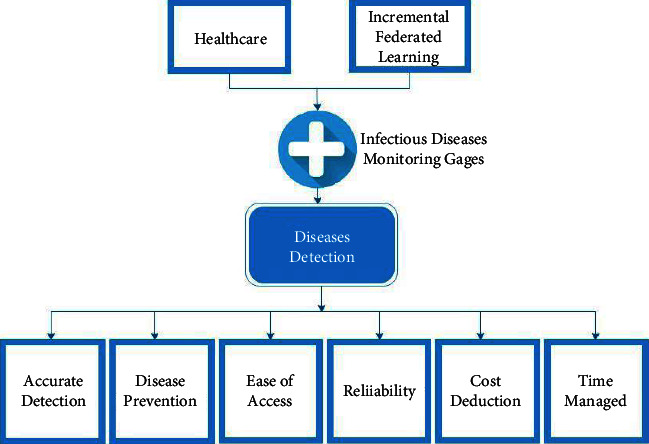
Use of infectious diseases monitoring gages.

Deep learning is currently used in the electronic health record to anticipate healthcare-associated illnesses and to minimize administrative load [7]. Medical practitioners focused on healthcare concerns as a result of reducing administrative difficulties and enhancing access to essential patient records [8]. The use of biomedical data in deep learning is becoming increasingly important in the age of healthcare. The use of electronic health records helps to make sure that the proper medication and prescription are provided to the persistent environment and molecular traits [9]. By learning about all infectious diseases and their cure, the right treatment can be given to the target patient. It is difficult to examine the symptoms of infection and identify which kind of infection the patient is suffering from. Deep learning can work for the detection of these diseases by using an efficient framework with the help of its effective learning feature [10].  Figure 1 shows the impact of using federated learning-based monitoring gages for the detection of infectious diseases.

This systematic study is designed to highlight different machine learning approaches, especially federated learning, for accurate detection. It highlights some future possibilities, which help to design different wearable gages for the early diagnosis of different infectious diseases. Different social media platforms are used for the detection of location of infectious diseases [11]. Through social media platforms, infectious diseases can be detected easily. For instance, messages from Weibo, Facebook, Instagram, WhatsApp, and Twitter have demonstrated their use as data sources for detecting and evaluating infectious illnesses [12]. Moreover, it thoroughly overlooks the architecture view of federated learning, which plays a vital role in mapping the local training data to centralized training master data [13]. For the execution of a systematic study, different research questions are designed to investigate general infectious disease monitoring games using a federated learning scheme. In this study, four electronic databases, ACM, IEEE Access, Springer, and ScienceDirect, are used to extract recent studies from 2018 to 2021. The extracted studies answer the RQs and how machine learning approaches are used for the recognition of different infectious diseases.

## 2. Materials and Methods

To detect infectious diseases with more effectiveness and accuracy, a systematic literature review is carried out. The best possible research questions are highlighted to support the research problem.

  RQ1: How do different machine learning algorithms play a vital role in the early identification of infectious diseases?
  RQ2: What is the robust impact of smart healthcare systems in recognition of different infectious diseases through distributed machine learning and deep learning models?
  RQ3: What is the influence of different federated learning models on the inclusion of the CNN (convolutional neural network) in the detection of infectious diseases?

### 2.1. Search Process

Different search strings are produced to search related studies. These search strings are then applied to find related results. These factors can then be used to improve healthcare systems by using the search string as a guide. Machine learning algorithms and smart healthcare systems can also be identified by using search queries. Various digital platforms are used to search for related studies. Google Scholar, IEEE digital library, and ACM are a few of them. To improve the accuracy of the search process, innovative strategies are implemented into the search strings.

(“Infectious diseases detection” OR “COVID-19”) AND (“Infectious disease recognition” OR “Infectious diseases classification”) AND (Machine learning algorithms”) AND (“Intelligent healthcare systems”) AND (“Distributed machine learning”) AND (“Federated learning in healthcare”).

### 2.2. Inclusion and Exclusion Criteria

Our study is primarily focused on healthcare and improving it by using machine learning techniques. To achieve this, the inclusion/exclusion criteria are established to obtain results that are related to the research problem.  Table 1 highlights the inclusion scheme of the collected studies, and Table 2 represents the exclusion scheme that supports the cleaning process.

### 2.3. Data Collection and Cleaning

**Figure 2:**
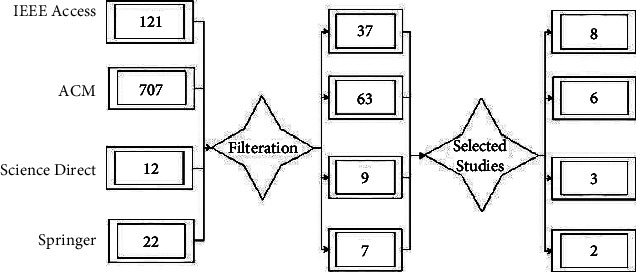
Study filtration and selection process.

**Figure 3:**
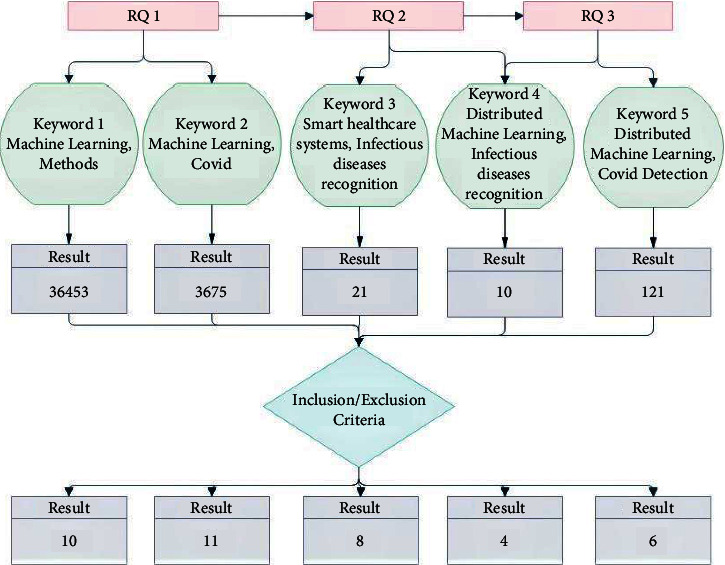
Structural diagram of the study extraction process.

There are plenty of ways to collect data, but electronic databases are the most used in the extraction of data. The data were extracted from four main electronic databases from the relevant literature. These electronic databases are IEEE, ACM, ScienceDirect, and Springer. Research questions are focused on data collection, with only relevant research studies added to support the questions. After applying the inclusion and exclusion criteria, the extracted studies are used to do a systematic literature review. The extracted literature supports our research problem, while Figures 2 and 3 support the data collection and cleaning process.

After different filtration schemes, 21 articles were extracted from databases and mapped to defined RQs, Table 3. Moreover, the highlighted mapping of fetched articles to RQs declares those parameters of the federated learning scheme, which help to design in the future in terms of monitoring infectious diseases wearable gages.

## 3. Discussion on Current Trends

In this section, a mapping of related work is carried out to discover how many selected papers are related to the research questions. These selected studies are discussed in the bibliometric analysis. The selected study covers all research questions about how machine learning is used for the recognition of infectious diseases.

### 3.1. Architectural View of Centralized Machine Learning Techniques

Deep learning models consist of increased volumes of unsupervised data to produce complex representations with greater accuracy than machine learning traditional approaches. Hierarchical learning is simulated by using artificial multilayer neural networks. This allows all layers to generate various attributes by using raw information. High-end machines are required for DL algorithms because they work with a large amount of data and provide advanced solutions [14]. As a result, deep learning relies heavily on the graphics processing unit. The feature extraction improves performance and decreases the data complexity in ML. Learning high-level functions and data without the manual input of domain experts is possible with deep learning algorithms [15]. In regard to the test phase, the deep learning algorithm is much faster than machine learning algorithms and provides more accurate results [16]. To identify solutions to complex health issues and provide patients with long-term treatment, the algorithms of ML and DL are applied [17]. Providers of healthcare can benefit from medical images by merging them with demographic data [18]. In addition to DNNs and RNNs, there are also probabilistic neural networks (PNNs) and feed-forward neural networks (FFNNs). Most DL systems use CNNs (ResNet and GoogleNet) and recurrent neural networks (RNNs) (LSTM and GRU (gated recurrent unit)). Stacking autoencoders is also popular in machine learning [19].

### 3.2. Convolutional Neural Network

The input, hidden, and output layers are regular neural network layers. This is because every layer contains neurons, and each neuron of the present layer is connected to a neuron of the previous layer, so all neurons are of high weightage. This method is effective in predicting simple and small data but fails when dealing with complex data objects and translations. Cells are only connected to their nearest neighbours in the convolutional layer, and all cells have the same weight.  Figure 3 highlights the structure of the CNN with the inclusion of input, output, and hidden layers.

**Figure 4:**
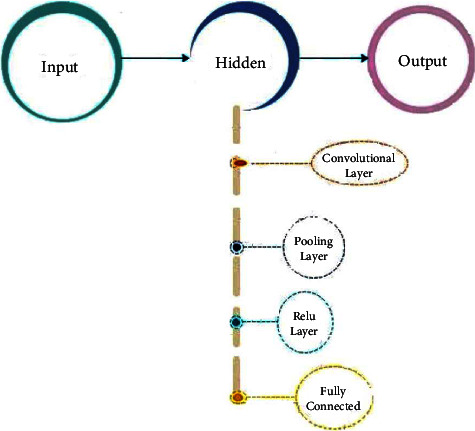
Structure of the convolutional neural network (feed-forward).

In the figure of the CNN, we will treat eyes as a separate object in image detection; it will not find eyes all over the image. The CNN requires images of a fixed size as an input, and preprocessing is required to achieve output. These key features are then stored in a database for preprocessing before they are sent to an application. Features of these images are detected and used for further image detection and classification.  Figure 4 shows the flow of the CNN. Layers such as the convolutional pooling and ReLU (rectified linear activation function) functions, as well as a fully connected layer, are all used to build the network. It is divided into several layers of kernels. Each kernel covers a specific feature of the object with specific dimensions. Kernel 1 will detect the eyes of the object, kernel 2 will detect the nose, kernel 3 will detect its lips, and kernel 4 will detect the shape of the object. Next-layer classification and accurate prediction will be based on these vectors [20].

Max or average algorithm is used for the feature map to decrease its range. This algorithm increases the speed of the pooling layer. The supreme area of a particular feature map is taken as input and places in the same area are returned as output in the max-pooling process. When using average pooling, a feature map of average size is used as input. Negative values are converted to zero in the ReLU layer. Using activation, classify the input into a fully connected layer and assign it a class score.

Infectious disease instances are detected with the help of an extremely basic CNN model. This model contains a single convolutional layer with sixteen filters. These filters are followed by the batch normalization layer, the ReLU layer, two fully connected layers, and the final layer, the SoftMax layer. A preprocessed picture dataset is read into the input layer of the model. These images are subjected to a separate preprocessing phase. Images are cropped and resized during the preprocessing stage. Primarily, the purpose of convolutional is to extract features from a picture dataset and establish a spatial connection between image pixels in the image. To decrease the number of training epochs required for deep network stabilization and training, a batch normalization layer is used. As a result of the use of the ReLU layer, the negative pixels in the convolved features are replaced by zeros. A nonlinearity map of CNN's features is generated by using this function. The primary job of the fully connected layer is to classify the recovered features from picture datasets into classes. The function of the Softmax layer is purely for determining the activation function results from the probability values of the preceding layer. In the diagnosis of infectious diseases, the values can be classified into two classes: “0” and “1.” In the last output layer of the CNN model, results from the previous layer can be labelled. Therefore, for instance, a COVID-19 value of “1” indicates a positive case, while a non-COVID-19 value of “0” indicates that the chest X-ray or CT was normal [21].

### 3.3. Recurrent Neural Networks

**Figure 5:**
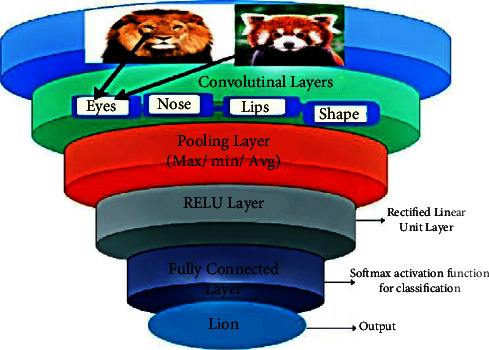
The internal flow of the CNN model.

Because of its memory, the RNN can analyse data sequences of variable length and store them in its database. In addition, it takes into account the previous input state [22]. When making predictions, it uses information from its past, and an infinite number of steps are repeated indefinitely to propagate information through its hidden state over time [23].  Figure 5 shows the structure representation of the RNN.

**Figure 6:**
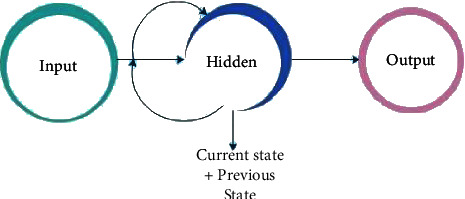
RNN structural diagram.

**Figure 7:**
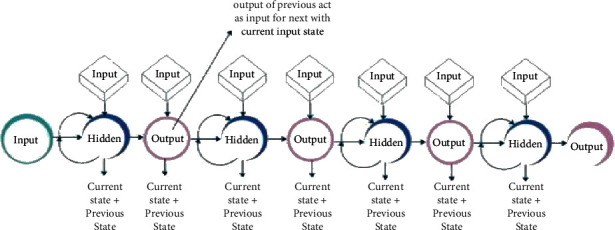
Sequential RNN model.

It manipulates current and recent past states to produce a new data output [24]. The output is used to determine the previous state for the next time step. RNNs have short-term memory because of this role. In addition to language generation and DNA sequence analysis, it is also used in text assessment, sound analysis, time string analysis, and many other applications because it is extremely efficient for data sequences that occur in time. A simple and robust RNN is a good model to use [25].  Figure 6 describes the internal flow of the RNN model. (See Figure 7)

Because the CNN only focuses on the current input state, it has no memory and is unable to handle sequential data [26]. It is, therefore, essential to employ an RNN model for the improvement of the prediction and to manage sequential records. Then, the RNN model feeds itself data by using the output as a previous state for the next time step. Data can be checked over time using RNNs [27].

### 3.4. Deep Neural Network

The layered architecture of advanced systems is used in DNN's architecture and implementation. Processing power and hardware performance are required for performing complex tasks. Models such as the DNN are used for classification and regression purposes. Classification results are more precise in complex classifications than the method itself [28]. For several years, DNNs were deemed impractical because they required too much computational power to train and process, for instance, real-time applications [29]. Due to advancements in hardware and synchronization by GPUs (graphics processing units) and big data, DNNs are now considered a major technological innovation in the field [30].

### 3.5. Probabilistic Neural Network

Feed-forward neural networks, such as PNNs, are commonly used to solve classification and pattern recognition concerns. A nonparametric function and a Parzen window approximate the PDF function for each class in the PNN. A PNN structure consists of 4 layers, an input pattern layer, as well as a summation and output layer.

The greatest operational advantage of the PNN is that the training is quick and easy. As soon as a pattern from each category is recognized, the network can begin generalizing to new patterns. As more patterns are discovered and saved in the network, the generalization improves, and the decision boundary becomes more complex.

### 3.6. Reinforcement Learning

**Figure 8:**
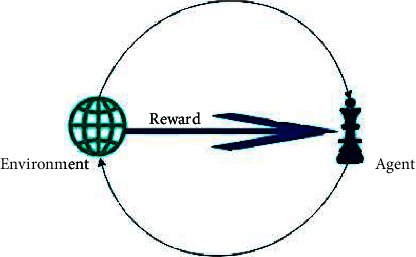
Reinforcement learning basic diagram.

In reinforcement learning, there is no way to predict the outcome, so the system must choose the best course of action. Reward-based learning is also called a behaviour-based process. In the reinforcement learning system, you receive a reward based on behaviour. Critics point out that the current situation is better than it was in the past.  Figure 8 represents the environment. Agent, reward, state, and action are the five components of a reinforcement learning agent [31].

To maximize the positive reward, reinforcement learning focuses on agents' intelligence. Reinforcement learning differs from supervised learning because, in supervised learning, there is no need for input or output labels. As such, it aims to strike a balance between previous and current information. Using techniques from dynamic programming, the environment acts like a Markov decision process [32].

In reinforcement learning, there is no way to predict the outcome, so the system must choose the best course of action. Reinforcement learning is behaviour based. In the reinforcement learning system, get the reward according to the behaviour of the object. Critic information shows the current state rewards concerning the past. There are five elements of reinforcement learning: agent, environment, reward, state, and action [33].

### 3.7. Architectural View of Federated Learning

**Figure 9:**
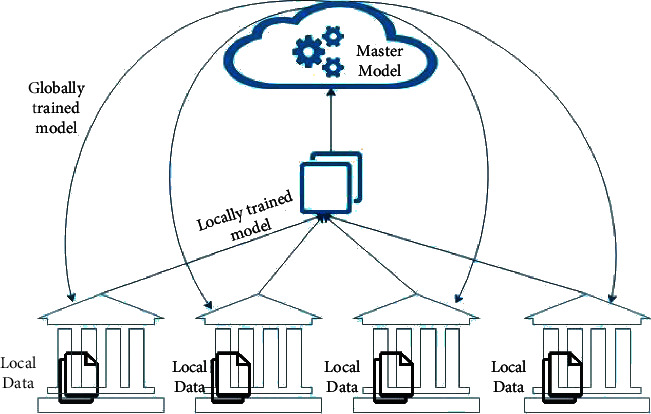
The architecture of federated learning.

A “federated learning” technique involves training an algorithm without exchanging information between servers containing local data samples or other clustered edge gadgets as compared to conventional centralized machine learning methods, in which all local datasets are transferred to a single server and trained using the master model that will further globally train the peer nodes [34]. Data access rights, data privacy, heterogeneous data access, and security are factors that can be addressed with the help of federated learning. Pharmaceutics, telecommunications, and IoT (Internet of Things) are among the industries where federated learning is used in effective applications [35].  Figure 9 represents the architecture of federated learning, which highlights the training of local data and synchronizes it with the master model of the ANN.

Without unambiguously trading samples of data, the goal of federated learning is multiple datasets stored in local nodes used to train machine learning algorithms. To create a linear model that is shared by all endpoints at some frequency, the models are trained locally using data samples collected locally [36].

More effective machine learning approaches can be used to improve smart healthcare systems. Using a distributed machine learning model to detect infectious diseases will provide more accurate and justified outcomes [37]. The disease detection systems or devices are lacking in quality and reliability; there is room for future research in distributed machine learning approaches to improve disease detection technology [38]. This will benefit the healthcare business as well as human health. Human life will be safeguarded by accurate predictions made at the appropriate moment and with good medical records.

## 4. Future Work: Incremental Federated Learning Model

In contrast to distributed learning, which maximizes computing power, federated learning focuses on training a dataset that is heterogeneous [39]. A widely known underlying assumption in distributed learning is whether the local datasets are identically distributed and the same size, even though it also aims to train a single model on multiple servers. For federated learning, these hypotheses are not applicable; rather than homogeneity, datasets tend to be heterogeneous and have a range in size. As a result of their dependence on ineffective communication media, clients who are participating in federated learning could be unpredictable battery-powered systems and wireless technology (IoT devices and smartphones), but in distributed learning, all nodes are used as data centers with advanced computing capabilities and high-speed network connections. Federated learning is a smarter model with a lower legacy and less power consumption.

These machine learning approaches are very efficient in detecting infectious diseases more accurately with their efficient algorithms and frameworks. Smart healthcare systems can further be upgraded by implementing more effective machine learning approaches. The detection of infectious diseases will give more accurate and justified results by using distributed machine learning approaches. These infectious diseases can include the detection of hepatitis (B or C), malaria, dengue, tuberculosis, and COVID-19 as well. The use of decentralized learning can make detection and prediction accurate and will be able to work with the latest data as well as old data. The framework of federated learning can be helpful in learning about decentralized data.

**Figure 10:**
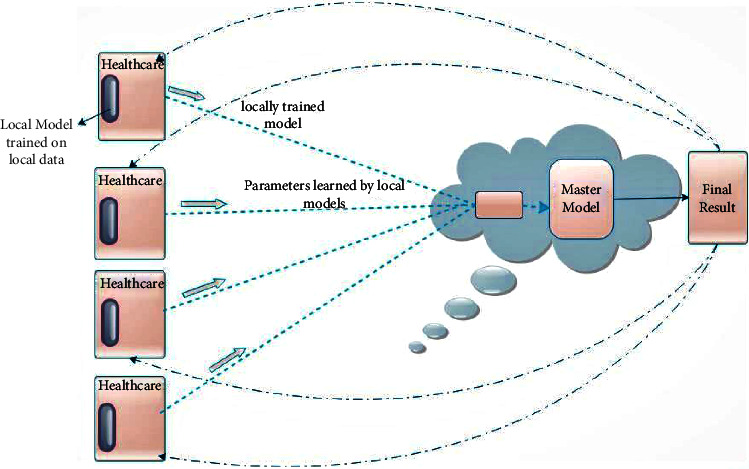
Incremental federated learning integrates into current digital healthcare systems.

In the future, smart healthcare systems can be upgraded for the recognition of different infectious diseases by using distributed federated learning clusters.  Figure 10 displays the next generation healthcare systems that will help to robustly recognize different infectious diseases. In distributed federated learning clusters, every smart healthcare system has locally trained a model for the prediction and recognition of different diseases. Moreover, the distributed federated learning clusters will take all parameters from these smart healthcare systems and generate a master model [40]. Such a master model will not take data for learning. Instead, it will take all parameters of smart healthcare systems and train itself through these parameters to generate a master model.

**Figure 11:**
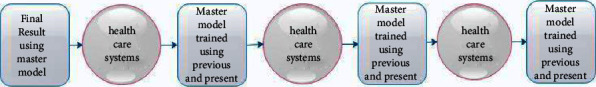
Healthcare model overview using distributed federated learning.

Furthermore, the master model will be the initial model of the next round, and at every round of training, the master model will learn more. This master model will have the training experience of models of all healthcare systems, so it will predict more accurately.  Figure 11 is a representation of the master model increment after every round.

The above-highlighted model can be improved with time and will predict more accurately. This distributed technology will get parameters from multiple healthcare system models. These systems will have a local model, and that local model will work with machine learning algorithms to predict the results. The parameters of these local models will be transferred to a decentralized master model. This master model will learn from all parameters and predict accordingly. This model will help to learn from the present and previous models. The local model will learn from the new data at every round, and then, the master model will learn from the parameters of the local model. The master model will also learn from the parameters of previous local models. Therefore, the use of a decentralized learning approach will be helpful in improving the performance of smart healthcare systems and the recognition of infectious diseases.

## 5. Conclusions

With the rapid advancement in the modern healthcare system, machine learning is used for the detection of infectious diseases. These healthcare systems play a vital role in the detection of infectious diseases, maintaining healthcare records, and in communication with doctors. The healthcare systems are giving the healthcare industry easy and more effective ways to cure and identify diseases. A systematic literature review is carried out to identify upgrades in smart healthcare systems. Kitchenham guidelines are followed to extract the literature from the study by using four electronic databases. Different technologies and machine learning algorithms are used in the detection of infectious diseases. These algorithms are working on centralized data for prediction, due to which it is difficult for healthcare systems to learn the latest data and to deal with the latest technologies with innovations. These machine learning approaches are very efficient in the more accurate detection of infectious diseases with their efficient algorithms and frameworks. Smart healthcare systems can further be upgraded by implementing more effective machine learning approaches. The use of decentralized learning can make detection and prediction accurate and will be able to work with the latest data as well as the old. As a result, a framework based on federated machine learning is introduced in this study. Wearable devices will be used to assist in the earlier detection of infectious diseases through federated learning. Federated learning is a smarter model with a lower legacy and less power consumption. Federated learning will be helpful in the precise detection of infectious diseases, which will also reduce the chance of death. The healthcare community will also be able to use it for the detection of COVID-19 and will work with the software industry to further improve it. The detection of infectious diseases will give more accurate and justified results by using distributed machine learning. These infectious diseases can include the detection of hepatitis (B or C), malaria, dengue, tuberculosis, and COVID-19 as well. Moreover, these gages will help to reduce the time and death rate by detection of severe diseases at starting stage. The accuracy and sustainability of the healthcare gadgets will be carried out by using these algorithms.

## Figures and Tables

**Table 1:** Inclusion scheme in the study collection.

No.	Inclusion criteria
1	Discuss the optimized methods of machine learning
2	Discuss the limitations of the use of distributed machine learning with the comparison of federated learning
3	Papers discuss the flow of federated learning in biomedical application
4	Papers discuss the detection of COVID-19 via deep learning models

**Table 2:** Exclusion scheme for the cleaning process.

No.	Exclusion criteria
1	Not the English language scholarly article.
2	Parameters of distributed machine learning are not defined clearly
3	Results are not clearly defined in biomedical applications
4	Parameters of federated learning are not defined clearly

**Table 3:** Bibliometric measurement.

Ref#	Key factors	Merits	Demerits	Mapping	Year
[2]	EHRs (electronic health records) are supplemented with hierarchical information from medical ontologies by using a GRAM (graph-based attention model)	GRAM is performing excellently. When the data are inadequate, it works well.	Improvement is needed in the way this method incorporates knowledge DAG (directed acyclic graph) into neural networks.	RQ2	2017

[7]	Restricted Boltzmann machines and autoencoder stacked units' networks are implemented	Comparing the results of deep learning methods, which have highly precise values	Experiment with a broader scope of preprocessing methods is needed.	RQ3	2016

[8]	To identify infectious disease host genes, a machine learning classification technique is developed	Wide-scale host gene prediction connected to infectious diseases is made possible.	There are no major benefits of not being able to use a small-scale dataset.	RQ1	2019

[12]	Comprehensive review study for the diagnosis of COVID-19 via deep learning models	A detailed review of different diagnosis methods of COVID-19 by using the different structures of the CNN and ResNet-50 (residual network-50) model.	Computational complexity factor requires to highlight clearly	RQ2	2019

[13]	Added recent research on the use of DL (deep learning) to improve the domain of health care.	Big biomedical data could be translated into improved human health by using deep learning techniques.	The development of applications needs to be improved.	RQ2, RQ3	2018

[10]	CNN is a perfect model to use for the analysis of applications and challenges of medical images.	It can detect infectious disease outbreaks, among other applications.	System inconsistencies include heterogeneity of data quality and security.	RQ2	2021

[14]	Use of neural networks in the prediction of diseases.	Helps to identify how neural networks can be helpful in detecting infectious diseases.	Results and technical parts are missing, which would be helpful in implementing the framework	RQ2	2019

[15]	Medical, e-healthcare, and bioinformatics applications of DL are discussed.	Contains effective DL methods for biomedical and health-related applications.	In healthcare, distinctions between deep learning technologies and techniques need to be improved.	RQ2	2020

[16]	SAPS II and SOFA ratings (severity scores) ML ensembles were compared for quality check.	As per the results, the DL model defeated most other techniques.	Current data must be added.	RQ2	2018

[17]	Privacy concerns are highlighted in the flow of EHR through federated learning.	A unique federated learning framework proposed for efficient diagnosis of different human diseases	At least discuss the computational complexity in the flow of HER through federated learning.	RQ3	2018

[18]	The fusion-based federated learning model for accurate detection of COVID-19.	Medical image analysis for detection of COVID-19 for better communication and performance if federated learning model.	Along with the accuracy factor, the robustness parameter is missed in the proposed model.	RQ3	2021

[19]	Deep learning techniques are used which are working in healthcare	Exposed a few key areas of medicine where DL computational methods can have a positive impact.	Some other techniques of deep learning are not discussed	RQ2	2019

[20]	Driver drowsiness is predicted by using a deep CNN model.	Helps to create an improved system that detects driver drowsiness by using the deep CNN	Needs further improvement in eye detection speed.	RQ1	2019

[21]	DeepSol, a novel protein solubility predictor based on deep learning, has been proposed by researchers.	DeepSol has overcome the limitations of its feature selection step and two-stage classifier.	It can be projected with DeepSol to lower costs.	RQ2	2018

[22]	FML (federated machine learning) thoroughly discusses the different parameters of training and testing the ML models.	A comprehensive review of the concepts of vertical and horizontal federated learning models. Moreover, we thoroughly discussed the applications of FML inclusion in healthcare applications.	Compromises detailed discussion on security protocols when electronic health records move from one node to another node.	RQ3	2019

[23]	An evolutionary algorithm is proposed for training a DNN (deep neural network) model for the estimation of morbidity of gastrointestinal infections.	Compared to the extensively used ANN (artificial neural network) and MLR (multiple linear regression) models, this model is much more accurate at predicting disease morbidity.	Further samples should be collected, and pollutants should be determined.	RQ2	2017

[24]	On the MovieQA question answering dataset, a model is presented.	Models are learning matching patterns for the selection of the right response.	To improve machine reading comprehension, the system should include entailments and answers.	RQ1	2018

[25]	This study introduced the independently recurrent neural network.	By learning long-term dependencies, IndRNN (independently recurrent neural network) helps to prevent gradient explosion and disappearance.	It is not possible to improve the performance of the LSTM (long short-term memory) by raising the size of parameters or layers.	RQ1	2018

[26]	The performance of ML networks is compared to that of feed-forward neural networks, also with logistic regression.	The XGB (gradient-boosted trees) model, which was found to be the most accurate, outperformed the logistic regression in terms of calibration.	There is a need for further research to improve the prediction of administrative information.	RQ1	2020

[27]	The RNN technique can be formally developed for differential equations by using the RNN canonical formulation.	Signal processing-based analysis of RNNs and vanilla LSTMs and comprehensive treatment of the RNN concepts using descriptive and meaningful notation are presented.	The augmented LSTM system is effective, but it needs to be enhanced with more techniques.	RQ1	2020

[28]	Developed a wearable body sensor fusion data-driven deep RNN activity recognition system.	A human's functionality and lifestyle can be determined based on physical actions by using body sensors.	A human behaviour monitoring system can further be evaluated in real-time on overly complex datasets.	RQ1	2020

## Data Availability

There is no data involved in the composition of this study.

## Abbreviations

RNN: Recurrent neural network
CNN: Convolutional neural network
PNN: Probabilistic neural network
DNN: Deep neural network
ML: Machine learning
DL: Deep learning
RL: Reinforcement learning
FL: Federated learning.
